# Expression patterns of circulating microRNA in confirmed early-onset and late-onset pre-eclampsia cases

**DOI:** 10.1007/s00404-026-08583-0

**Published:** 2026-09-28

**Authors:** Isabel Hoffmann, Jochen Maurer, Christian Enzensberger, Elmar Stickeler, Oliver Graupner

**Affiliations:** Department of Obstetrics and Gynecology, University Hospital Aachen, RWTH Aachen University, Pauwelsstraße 30, 52074 Aachen, Germany

**Keywords:** microRNA, Pre-eclampsia, Placenta, Angiogenesis, Anti-angiogenic imbalance

## Abstract

**Purpose:**

The aim of our study was to investigate selected pre-eclampsia (PE)-related miRNAs in clearly defined early- and late-onset PE (eoPE, loPE) cohorts and compare them with healthy controls.

**Methods:**

This is a single-center, pilot study comparing pregnancies with eoPE (n=10) and loPE (n=8), each with separate healthy controls (n=10; n=8). Serum miRNA (miR-) expression levels of selected PE-associated miRNAs (miR-210-3p, miR-518b) were quantified by real-time PCR.

**Results:**

Similar expression profiles (delta CT ± standard deviation) were observed between loPE vs. controls: miR-210-3p (8.20 ± 1 vs. 7.48 ± 1.2; p=0.16; Hodges-Lehmann estimator (HL): − 0.73, 95% confidence interval (CI) − 2.2 to 0.78) and miR-518b (9.82 ± 1.1 vs. 9.60 ± 0.9; p=0.80, HL: − 0.14, 95% CI − 1.61 to 0.91). Comparing eoPE cases to controls, we found an increase of miR-210-3p concentration (7.63 ± 1.1 vs. 9.31 ± 1.9; p=0.019; HL: 1.23, 95% CI 0.15 to 2.66, mean fold change: 3.2; sensitivity analysis: p=0.067) and a numerical difference in miR-518b expression without reaching statistical significance (8.77 ± 1.4 vs. 9.85 ± 1.8; p=0.19; HL: 0.70, 95% CI − 0.33 to 2.41; mean fold change: 2.1).

**Conclusion:**

In the eoPE, miR-210-3p levels were higher than in controls, with the same trend observed in the sensitivity analysis, although statistical significance was no longer reached (p=0.067). miR-518b showed a numerical, non-significant difference between eoPE and controls. No significant differences were observed in loPE. Given the small sample size and overlap with early-onset fetal growth restriction, these findings should be considered exploratory.

## What does this study adds to the clinical work

**Table:** 

This pilot study provides preliminary observations on circulating miR-210-3p and miR 518b expression in eoPE. Given the small sample size and potential confounding factors, including hypertension, diabetes, and eoFGR, the findings are exploratory and do not establish clinical utility. Larger, well-characterized studies are needed to determine whether these observations can be confirmed.	

## Introduction

Pre-eclampsia (PE) affects 2–5% of pregnancies and is a leading cause of maternal and fetal morbidity and mortality worldwide [1]. PE is a multisystem, hypertensive disorder characterized by endothelial damage in multiple organs, affecting both the mother and the fetus [2]. The exact pathophysiology of PE remains unclear. Current theories of PE pathogenesis discuss a dysfunctional maternal cardiovascular adaptation and a placental (anti-) angiogenic imbalance [3, 4]. PE is divided into two forms: eoPE and loPE, which manifest before 34+0 weeks or after 34+0 weeks. (Anti-) angiogenic factors, namely the sFlt-1/PlGF ratio measured in maternal blood, have been shown to improve the prediction of eoPE and loPE [5]. sFlt-1 (soluble Fms-like tyrosine kinase−1) and PlGF (placental growth factor) are both proteins produced in the placenta [6]. Circulating microRNAs (miRNAs) are known to affect placental function [7]. They play key roles in angiogenesis, cell proliferation, and differentiation as well as in inflammatory processes [8, 9]. miRNAs are non-coding, single-stranded RNAs, consisting of 20–24 nucleotides. They regulate gene expression by directing a splicing complex to a target RNA, resulting in an altered translation process and therefore decreased protein biosynthesis [10, 11]. The human placenta expresses up to 600 different miRNAs, which are influenced by environmental and maternal factors as well as intrauterine hypoxia [12, 13]. miRNAs produced in the placenta can be released into maternal circulation while being packaged in exosomes, which are formed by trophoblasts [14]. Altered miRNA expression patterns in maternal blood or the placenta itself have been found in placenta-related diseases such as fetal growth restriction (FGR) and/or PE [15]. In analogy to the 2-stage model of PE (stage 1: placental stage or impaired placentation; stage 2: maternal stage or release of numerous biological factors causing endothelial dysfunction), several miRNAs have increased or decreased concentrations in maternal serum or the placenta [15–17].

Two miRNAs, miR-210 and miR-518b, are frequently reported across studies to be elevated in placental or blood samples from PE patients. miR-210 is the best-studied miRNA in PE. As a hypoxia-responsive miRNA, it plays an important role in angiogenesis, trophoblast proliferation, and differentiation [18, 19]. Upregulation of miR-210 has been found in both placental and maternal blood samples from PE patients [15].

miR-518b belongs to the chromosome 19 miR-cluster (C19MC), which is increasingly expressed in placental trophoblast cells. The cluster is involved in placentation, so its dysregulation may contribute to the pathogenesis of PE [20]. miR-518b interacts with the EGR-1 protein (early growth response protein 1), a zinc-finger transcription factor that plays an important role in angiogenesis, cell differentiation, and migration [21]. Dysregulation of miR-518b has been shown to decrease EGR1 and VEGF (vascular endothelial growth factor), leading to inhibition of trophoblast cell migration and angiogenesis [21].

Accordingly, miRNAs are increasingly discussed as potential biomarkers of PE, although the cause-and-effect relationship has not yet been clarified. However, one main criticism, in our opinion, of previous studies on the role of miRNA in PE is the heterogeneity of PE cohorts in terms of gestational age (GA) (early <34+0 vs. late ≥34+0 weeks) at sample acquisition [12]. Therefore, the aim of our study was to examine circulating miRNA expression levels in a well-defined cohort of eoPE and loPE to compare them with those in healthy controls.

## Materials and methods

### Study population

We conducted a single-center pilot study with a cohort of women with eoPE with GA-matched healthy pregnant controls (mean GA 28+2 weeks vs. 30+6 weeks, n=20) and a separate cohort of women with loPE and corresponding GA-matched healthy controls (mean GA 37+0 weeks vs. 37+0 weeks, n=16). All eoPE cases delivered before 34+0 weeks and developed severe features during pregnancy, according to national (AWMF) and international guidelines (ISSHP/NICE) [22–24]. 80% of our included eoPE cases also had an associated early-onset fetal growth restriction (eoFGR). Exclusion criteria were age <18 years, fetal anomalies and/or chromosomal aberrations, and serious maternal diseases.

PE was defined according to national guidelines [24]: Elevated blood pressure (systolic 140 mmHg and/or diastolic 90 mmHg, on two occasions at least 4 h apart) and one of the following: proteinuria (300 mg/24 h), thrombocytopenia (platelet count <100.000/µl), poor liver function (elevated blood levels of liver transaminases to twice the normal concentration), new-onset renal insufficiency (elevated serum creatinine ≥0,9 mg/dl), pulmonary edema, new-onset cerebral or visual disturbances, FGR, and/or sFlt-1/PIGF >85/>110. eoFGR was defined according to the ISUOG guidelines [25]: abdominal circumference (AC) and/or estimated fetal weight (EFW) <3rd centile, or absent end-diastolic flow (AEDF) in an umbilical artery (UA) Doppler, or at least two out of three of the following:

1. AC/EFW <10th centile,
2. Uterine artery pulsatility index (UtA-PI) >95th centile,
3. UA pulsatility index (PI) >95th centile.

According to our clinical protocol and national guidelines [24], pregnancies with loPE were delivered electively by induction of labor (or by elective cesarean delivery if there was a medical indication) from 37+0 weeks onward or earlier in case of severe features. The indication for delivery in the cohort of eoPE was based on maternal or fetal clinical condition and severe features [24]. Data on PE-related adverse perinatal outcomes were based on the Delphi consensus [26].

### Selection of miRNA and Sampling

The expression levels of two frequently reported PE-associated miRNAs (miR-210-3p and miR-518b) were examined [15]. In total, one serum probe containing 7.5 ml of whole blood was collected from each mother at the time of PE diagnosis. The serum samples were processed as previously described [27]: samples were stored until fully coagulated, then centrifuged at 2500 × g for 10 min. Then serum supernatant was carefully pipetted and divided into aliquots. Samples were stored at − 80 °C and isolated in subsequent steps.

### RNA isolation, reverse transcription and amplification

RNA isolation was performed using miRNeasy Mini Kit (Qiagen, #217004) according to the user manual. Aliquots contained each 2µL of serum each or isolation and were eluted in 1μL of RNA-free water. After isolation, the RNA was stored at − 80 °C. To synthesize cDNA, the TaqManTM Advanced cDNA Synthesis Kit (#A28007, Applied BiosystemsTM) was used. 2μL of RNA was used for transcription. The synthetic ath-miR-159a was included as a spike-in control to ensure accurate amplification. cDNA samples were stored at − 20 °C until subsequent RT-qPCR analysis.

### Real-time qPCR

Samples were diluted 1:10 with 0.1X TE buffer (1xTE pH 8.0 from PanReac AppliChem (A2575)) as recommended by the manufacturer’s instructions. Transcribed miRNA samples were analyzed using the TaqMan Advanced miRNA Assay (#A25576 Applied Biosystems) in combination with TaqMan Fast Advanced Master Mix (#4444557 Applied Biosystems). cDNA samples and the master mix were applied to 384-well plates (#04729749001 LightCycler 480 Multiwell Plate white, Roche) and pipetted in triplets (technical replicates) per miRNA assay. Assays always included duplets of the synthetic control ath-miR-159a, which was used as a reference gene in the statistical analysis. The Roche LightCycler 480 Instrument II (#05015243001) was used for detection.

### Statistical analysis

Data from the LightCycler 480 were exported as MS-EXCEL files and analyzed using GraphPad Prism 10. Resulting Ct (Cycle-Time) Values were analyzed using the ΔCt method. Because raw Ct values are influenced by differences in input RNA quantity and reverse transcription efficiency, normalization is essential for comparisons between samples. ath-miR-159a was used as a reference gene. This synthetic miRNA spike-in is absent in the biological samples and added in equal amounts to each specimen before extraction, thereby controlling for extraction efficiency and inter-sample variability. The ΔCt value was calculated as ΔCt = Ct(gene of interest)—Ct(housekeeping gene). As qPCR amplification is exponential, each unit difference in ΔCt corresponds to a twofold difference in relative miRNA expression. Consequently, a lower Ct value indicates earlier detection by the LightCycler 480 and therefore a higher miRNA concentration in the sample. Mean Fold-change was calculated using the 2^(− ΔΔCt) method. Statistical comparisons were performed directly on ΔCt values, as these approximate a normal distribution, unlike the derived linear fold-change values. Mean ΔCt values were calculated from technical replicates before further analysis. For statistical analysis, we carried out the Mann–Whitney U test. Given the exploratory nature of this pilot study and the limited sample size, the Hodges-Lehmann estimator (HL) and corresponding 95% confidence intervals (CI) were reported alongside p-values. The Hodges–Lehmann estimator was calculated as: control—PE. Therefore, positive Hodges–Lehmann estimates indicate higher ΔCt values in controls, whereas negative estimates indicate higher ΔCt values in the PE group. Since ΔCt was calculated as Ct(gene of interest) – Ct(housekeeping gene), lower ΔCt values correspond to higher relative miRNA expression.

We compared cases with eoPE (n=10) and loPE (n=8) with GA-matched healthy controls (n=10; n=8). Bonferroni correction for multiple testing (k=2) was applied separately within each independent cohort for the two investigated miRNAs (adjusted significance threshold: a = 0.025).

Although controls were GA-matched, a residual difference of approximately 2.4 weeks remained between the eoPE and controls. Therefore, GA was included as a covariate in an analysis of covariance (ANCOVA), performed with SPSS. Additional analyses were performed to evaluate the potential influence of clinical confounders. In the loPE cohort, GA, hypertension, and diabetes were included as covariates in the general linear model. In the eoPE cohort, hypertension and gestational diabetes were present only in the PE group and could therefore not be included as covariates. Instead, we conducted a sensitivity analysis excluding these two patients, both of whom had pre-existing hypertension and gestational diabetes.

## Results

**Fig. 1:**
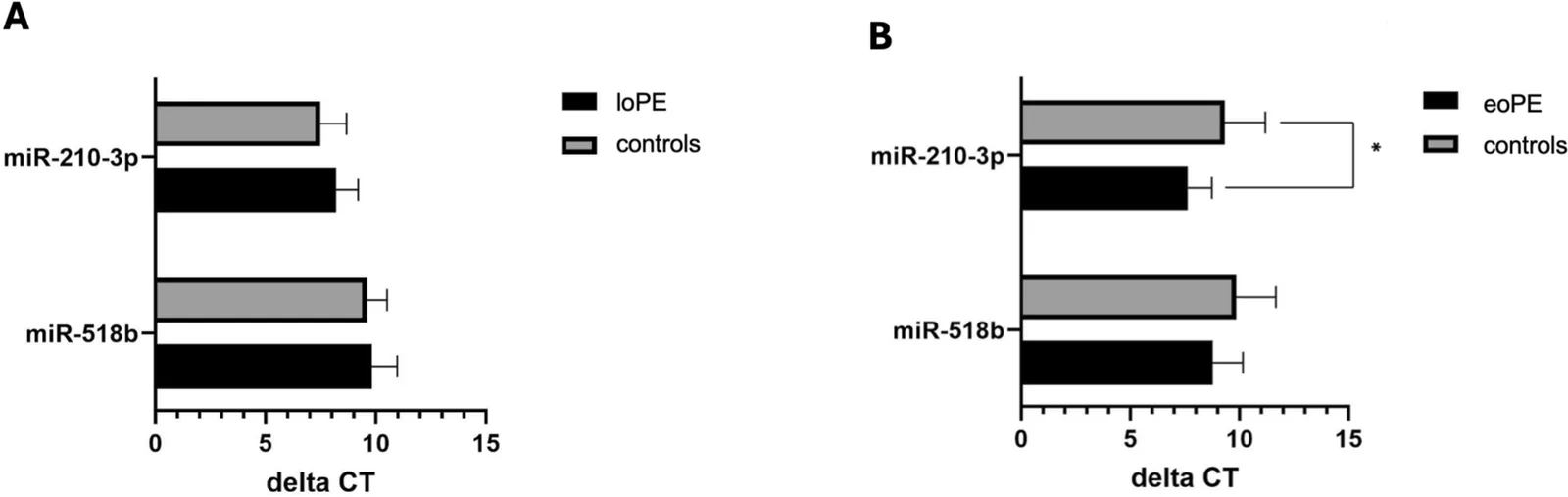
miR-210-3p and miR-518b mean concentrations (delta CT) **A** loPE (n=8) vs. controls (n=8), **B** eoPE (n=10) vs. controls (n=10); *: p<0.05; the lower the delta CT value, the more miRNA is expressed in the blood sample

Baseline characteristics of PE cases and perinatal maternal and fetal PE-related outcomes are presented in Tables 1, 2. sFlt-1/PIGF was determined at the time of PE diagnosis. Important Doppler sonographic values of the fetal and maternal vessels and the estimated weight were collected around the time of blood sample collection (Table 3). The following mean concentrations (delta CT) were detected in the loPE cohort vs. controls: miR-210-3p (8.20 ± 1 vs. 7.48 ± 1.2; p=0.16; HL: − 0.73, 95% CI − 2.2 to 0.78) and miR-518b (9.82 ± 1.1 vs. 9.60 ± 0.9; p=0.80; HL: − 0.14, 95% CI − 1.61 to 0.91) (Fig. 1A). The spike-in ath-miR-159a showed a mean CT value of 17.71 ± 0.26 (range 16.85–18.51).

**Table 1:** Maternal mean values of baseline characteristics for eoPE and loPE cases and GA-matched healthy controls, *GA* gestational age, *Inclusion-to-delivery interval*: days from study inclusion to delivery

	eoPE (n=10)	Early-onset Controls (n=10)	loPE (n=8)	Late-onset Controls (n=8)
GA inclusion (weeks + days)	28+2 (± 2.7)	30+6 (± 3.2)	37+0 (± 1.5)	37+0 (± 3.2)
Aspirin prophylaxis	3 (30%)	1 (10%)	0 (0%)	0 (0%)
Nullipara	7 (70%)	5 (50%)	5 (62.5%)	5 (62.5%)
sFlt-1/PlGF >85/>110	10 (100%)	/	5 (62.5%)	/
Inclusion-to-delivery interval (days)	16.8 (± 16)	61.7 (± 24)	7.3 (± 10.3)	23.3 (± 19.5)
GA delivery (weeks + days)	30+5 (± 2.7)	39+4 (± 1.4)	38+1 (± 0.8)	39+6 (± 0.6)
Severe features	10 (100%)	0 (0%)	5 (62.5%)	0 (0%)
BMI (kg/m^2^)	33.5	26.9	30.1	25.5
Smoker	0 (0%)	0 (0%)	0 (0%)	0 (0%)
Chronic Hypertension	2 (20%)	0 (0%)	3 (37.5%)	0 (0%)
Diabetes (preexisting)	0 (0%)	0 (0%)	1 (12.5%)	0 (0%)
Diabetes (gestational)	2 (20%)	0 (0%)	2 (25%)	1 (12.5%)

**Table 2:** Fetal and maternal core outcomes of eoPE and loPE cases with GA-matched healthy controls based on the published core outcome set [26]; *GA delivery:* gestational age at the time of delivery in weeks+days; *BE:* base excess; *NICU:* neonatal intensive care unit

	eoPE (n=10)	Early-onset Controls (n=10)	loPE (n=8)	Late-onset Controls (n=8)
GA delivery (weeks + days)	30+5 (± 2.7)	39+4 (± 1.4)	38+1 (± 0.8)	39+6 (± 0.6)
Neonatal body weight (gram)	1130 (± 345)	3546 (± 282)	3334 (± 590)	3778 (± 640)
5 Min. APGAR	8 (± 1)	10 (± 0.4)	10 (± 0)	9 (± 1)
Umbilical Artery pH	7.28 (± 0.06)	7.26 (± 0.07)	7.23 (± 0.05)	7.20 (± 0.04)
Umbilical Artery BE (mmol/l)	−1.54 (± 2.06)	−3.13 (± 2.24)	−3.8 (± 2.6)	−5.1 (± 2.6)
Neonatal acidosis	1 (10%)	0 (0%)	0 (0%)	0 (0%)
Admission to NICU	10 (100%)	1 (10%)	1 (12.5%)	2 (25%)
Neonatal respiratory support	10 (100%)	0 (0%)	0 (0%)	1 (12.5%)
Neonatal death	0 (0%)	0 (0%)	0 (0%)	0 (0%)
Neonatal seizures	0 (0%)	0 (0%)	0 (0%)	0 (0%)
Stillbirth	0 (0%)	0 (0%)	0 (0%)	0 (0%)
Maternal pulmonary oedema	1 (10%)	0 (0%)	0 (0%)	0 (0%)
Maternal placental abruption	1 (10%)	0 (0%)	0 (0%)	0 (0%)
Maternal postpartale haemorrage	0 (0%)	0 (0%)	3 (37.5%)	0 (0%)
Maternal raised liver enzymes	2 (20%)	0 (0%)	5 (62.5%)	0 (0%)
Maternal low platelets	4 (40%)	0 (0%)	4 (50%)	0 (0%)

**Table 3:** Doppler sonographic parameters for eoPE and loPE cases and GA-matched controls were assessed at the time of blood sample collection, therefore, they were linked to PE diagnosis

	eoPE (n=10)	Early-onset Controls (n=10)	loPE (n=8)	Late-onset Controls (n=8)
Mean EFW (gram)	983 (± 318)	2000 (± 790)	3006 (± 555)	3044 (± 644)
Mean EFW percentile	12 (± 20)	49 (± 17)	57 (± 34)	65 (± 23)
UA PI >95. P	5 (50%)	0 (0%)	0 (0%)	0 (0%)
UA AEDF	1 (10%)	0 (0%)	0 (0%)	0 (0%)
UA REDF	0 (0%)	0 (0%)	0 (0%)	0 (0%)
ACM PI <5. P	2 (20%)	0 (0%)	0 (0%)	0 (0%)
CPR <5. P	7 (70%)	0 (0%)	1 (12.5%)	0 (0%)
mUtA PI >95. P	8 (80%)	1 (10%)	1 (12.5%)	1 (12.5%)
Bilateral notching	7 (70%)	0 (0%)	0 (0%)	0 (0%)
DV-PIV >95.P	1 (10%)	0 (0%)	0 (0%)	0 (0%)
DV a-wave positive	10 (100%)	10 (100%)	8 (100%)	8 (100%)

*EFW* estimated fetal weight, *UA PI* umbilical artery pulsatility index, *UA AEDF* absent umbilical arterial end-diastolic flow, *UA REDF* reversed end-diastolic flow in the umbilical artery, *ACM PI* middle cerebral artery pulsatility index, *CPR* cerebroplacental ratio, *mUtA PI* maternal uterine artery pulsatility index, *DV-PIV* ductus venosus pulsatility index for veins, *P* percentile

**Fig. 2:**
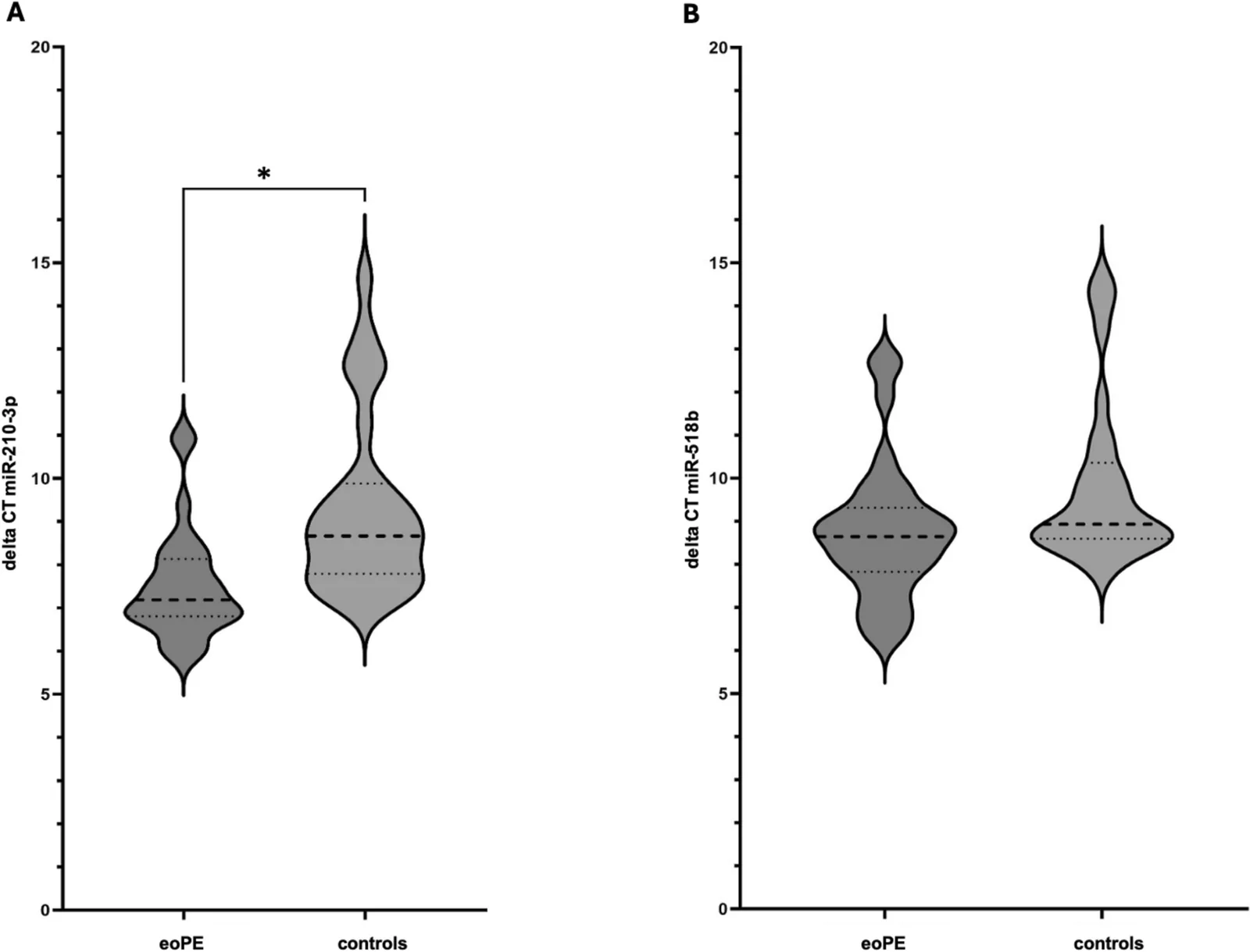
Mean miRNA concentrations (delta CT) in eoPE (n=10) compared to controls (n=10), showing the variability in miRNA expression patterns **A** eoPE vs. controls miR-210-3p, **B** eoPE vs. controls miR-518b; *: p<0.05, the lower the delta CT value, the more miRNA is expressed in the blood sample

In the eoPE cohort, we found an increase of miR-210-3p in PE cases in comparison to GA-matched controls (7.63 ± 1.1 vs. 9.31 ± 1.9; p=0.019, a=0.025, HL: 1.23, 95% CI 0.15 to 2.66; mean fold change: 3.2) and a numerical, non-significant, difference in miR-518b expression levels between eoPE cases and controls (8.77 ± 1.4 vs. 9.85 ± 1.8; p=0.19; HL: 0.70, 95% CI − 0.33 to 2.41; mean fold change: 2.1) (Fig. 1B). The spike-in ath-miR-159a showed a mean CT value of 17.75 ± 0.38 (range 17.04–18.9). When comparing the mean concentrations of both miRNAs in the eoPE group with those in controls, we observed variability within each group and overlap between groups (Fig. 2).

The ANCOVA did not provide evidence of a statistically significant association between GA and the expression of miR-210-3p (p>0.05) or miR-518b (p>0.05) in this small cohort of eoPE vs. controls. In the adjusted analysis of the loPE cohort, none of the included covariates (hypertension, diabetes, and gestational age) showed statistically significant associations with miR-210-3p or miR-518b (p>0.05). Sensitivity analysis in eoPE excluding two patients, both with chronic hypertension and gestational diabetes, showed a comparable direction and pattern of the observed differences, although statistical significance was no longer reached (p=0.067).

## Discussion

Our results may reflect prevailing theories of the pathophysiology of the two forms of PE. These theories hold that eoPE is caused by various stressors, the most important of which is uteroplacental malperfusion due to impaired remodeling of spiral arteries. Oxidative stress of the syncytiotrophoblast is another stressor that promotes the development of eoPE. In contrast, loPE is thought to be more due to interactions between normal placental senescence and maternal (genetic) predisposition to cardiovascular and metabolic disease [28, 29]. In addition, maternal factors such as obesity and significant weight gain during pregnancy are also thought to play an important role in the development of loPE [30]. New studies are raising further debate regarding the etiology of PE. These indicate that it’s not so much impaired remodeling of the spiral arteries that is the root cause, but rather the mother's cardiovascular maladaptation/pregestational cardiovascular dysfunction, which in turn results in impaired placentation with subsequent ischemia [28].

To support early diagnosis of PE, several miRNAs have been proposed as potential biomarkers. In different studies, an increase of miR-210 and miR-518b (among other miRNAs) in maternal circulation and the placenta has been reported [15].

However, to our knowledge, no clear distinction between eoPE and loPE has been made in previously published studies, which seems relevant for interpreting expression patterns in the context of the related anti-angiogenic imbalance and the degree of placental dysfunction [12]. Therefore, we recruited clearly defined eoPE and loPE groups.

In serum samples, mean miR-210-3p levels were threefold higher in eoPE patients compared to controls (7.63 ± 1.1 vs. 9.31 ± 1.9; p=0.019, HL: 1.23, 95% CI 0.15 to 2.66; mean fold change: 3.2), whereas loPE cases showed no difference for either miR-210-3p or miR-518b compared to controls. In eoPE, mean miR-518b levels were twofold higher than in controls, a numerical, non-significant difference (8.77 ± 1.4 vs. 9.85 ± 1.8; p=0.19; HL: 0.70, 95% CI − 0.33 to 2.41; mean fold change: 2.1). The sensitivity analysis excluding the two patients, both with chronic hypertension and gestational diabetes in the eoPE cohort, showed a comparable direction and pattern of the observed differences, although statistical significance was no longer reached (p=0.067). These findings should therefore be considered exploratory, and larger studies are needed to further investigate the two miRNAs as potential biomarkers. No differences were observed in the loPE group relative to controls.

The absence of significant differential expression in loPE suggests that neither miR-210-3p nor miR-518b is a suitable biomarker.

Also, there was an overlap in miR-210-3p and miR-518b expression levels between eoPE and the control group, suggesting that these findings should be interpreted carefully. This indicates that differences in miRNA patterns may not be enough to reliably differentiate eoPE patients from healthy pregnant women on an individual basis. Without a clear discriminatory threshold, the clinical use of these miRNAs as standalone diagnostic biomarkers is limited. Furthermore, the extent of miRNA concentration changes during pregnancy and in PE remains unknown. It is only established that miRNA concentrations change during pregnancy [31]. Here, further studies are needed to investigate potential reference values for miRNA concentrations across gestation. In our eoPE group, GA could not be matched exactly, resulting in a mean difference of 2.4 weeks between controls and eoPE cases. We performed an additional adjusted analysis using a general linear model, which didn’t provide evidence of a statistically significant association between GA and the expression of both miRNAs. However, given the limited sample size of this pilot study, the whole analyses should be considered exploratory.

Since miR-210-3p is a hypoxia-responsive miRNA, it is considered as a marker of uteroplacental hypoxia [19]. Awamleh et al. demonstrated increase of miR-210-3p in placental samples from patients with eoPE, FGR, and eoPE+FGR. Histopathological similarities between eoPE and FGR further support their shared placental pathophysiology [32]. This suggests that miR-210 reflects underlying placental hypoxia rather than PE-specific mechanisms. Accordingly, 80% of our eoPE patients, who delivered before 34+0 weeks and developed severe features during pregnancy, also presented with eoFGR. Other known causes of FGR (chromosomal abnormalities, viral infections) were not present in our patient cohort. This suggests that our findings cannot differentiate whether the observed increase in circulating miR-210-3p is primarily related to eoPE, eoFGR, or the underlying placental dysfunction. Future studies including isolated eoFGR are needed to clarify these associations.

Our study has several limitations. The most important limitation is the small sample size, which restricts statistical power and limits precise interpretation of miRNA expression levels. Therefore, caution is needed when interpreting these findings, and larger cohorts are needed to confirm the potential of miRNAs as reliable biomarkers. Also, a few patients in both PE cohorts had comorbid preexisting hypertension or gestational diabetes. Because miR-210 is reported to be increased not only in PE but also in hypertension and potentially in gestational diabetes, the specificity of these findings for PE remains uncertain [33, 34]. In the eoPE cohort, sensitivity analysis showed a comparable direction and pattern of the observed differences, though statistical significance was not achieved (p=0.067). No significant association was found between GA and miRNA changes. These results should be interpreted with caution due to the limited sample size. Residual confounding cannot be ruled out.

An endogenous reference miRNA was not included, which is a methodological limitation. As an exploratory pilot study, no prior sample size calculation was performed. The absence of a comparator group with isolated eoFGR prevents differentiation between the effects of eoPE and concurrent placental dysfunction. In addition, we did not examine all PE-associated miRNAs reported in the literature; instead, we focused on two frequently reported miRNAs. Finally, we have no clinical follow-up data, particularly regarding maternal cardiovascular outcomes.

The main strength of our study are the clearly defined eoPE and loPE populations based on diagnostic criteria from national and international guidelines, including established anti-angiogenic PE markers such as the sFlt-1/PlGF-ratio. Blood sampling was timed to sFlt-1/PlGF and Doppler assessment, allowing miRNA levels to be interpreted alongside established clinical markers of placental function. The focus on miRNA analysis in maternal circulation at the time of diagnosis of eoPE and loPE also appears advantageous, as, in our opinion, it better reflects a biomarker's potential for everyday clinical practice than postpartum testing of placental tissue.

## Conclusion

In conclusion, miRNA expression profiles were similar between loPE cases and controls. In the eoPE group, miR-210-3p levels were higher than in controls, with the same trend in the sensitivity analysis, although statistical significance was not maintained (p=0.067). miR-518b showed a numerical, non-significant difference. Given the small sample size and overlap with eoFGR, these findings are exploratory and require validation in larger studies.

## Data Availability

No datasets were generated or analysed during the current study.
